# Antiviral Effects of Animal Toxins: Is There a Way to Drugs?

**DOI:** 10.3390/ijms23073634

**Published:** 2022-03-26

**Authors:** Yuri Utkin, Andrei Siniavin, Igor Kasheverov, Victor Tsetlin

**Affiliations:** 1 Department of Molecular Neuroimmune Signaling, Shemyakin-Ovchinnikov Institute of Bioorganic Chemistry, Russian Academy of Sciences, 117997 Moscow, Russia; andreysi93@ya.ru (A.S.); shak_ever@yahoo.com (I.K.); victortsetlin3f@gmail.com (V.T.); 2 N.F. Gamaleya National Research Center for Epidemiology and Microbiology, Ivanovsky Institute of Virology, Ministry of Health of the Russian Federation, 123098 Moscow, Russia

**Keywords:** animal venom, antiviral activity, antiviral drug, bee, scorpion, snake, spider, virus

## Abstract

Viruses infect all types of organisms, causing viral diseases, which are very common in humans. Since viruses use the metabolic pathways of their host cells to replicate, they are difficult to eradicate without affecting the cells. The most effective measures against viral infections are vaccinations and antiviral drugs, which selectively inhibit the viral replication cycle. Both methods have disadvantages, which requires the development of new approaches to the treatment of viral diseases. In the study of animal venoms, it was found that, in addition to toxicity, venoms exhibit other types of biological activity, including an antiviral one, the first mention of which dates back to middle of the last century, but detailed studies of their antiviral activity have been conducted over the past 15 years. The COVID-19 pandemic has reinforced these studies and several compounds with antiviral activity have been identified in venoms. Some of them are very active and can be considered as the basis for antiviral drugs. This review discusses recent antiviral studies, the found compounds with high antiviral activity, and the possible mechanisms of their action. The prospects for using the animal venom components to create antiviral drugs, and the expected problems and possible solutions are also considered.

## 1. Introduction: A Brief Insight into Viruses and Viral Infections

A virus is a non-cellular infectious agent that can only reproduce inside cells. Viruses infect all types of organisms, from plants and animals to bacteria and archaea [1]. The genomes of viruses can be represented by DNA or RNA, in both cases being either single-stranded or double-stranded. A mature viral particle, known as a virion, consists of a nucleic acid surrounded by a protective protein coat called a capsid. The capsid is made up of identical protein subunits called capsomeres. Viruses may also have a lipid envelope over the capsid (supercapsid) formed from the membrane of the host cell. Viruses show a huge variety of shapes and sizes and as a rule, viruses are much smaller than bacteria. Most of the studied viruses have a diameter ranging from 20 to 300 nm.

Viruses do not have a cellular structure; therefore, they do not reproduce by cell division. Instead, they use the resources of the host cell to make multiple copies of themselves, and their assembly can occur at the cell membrane but also in exosomes or liposomes. Conventionally, the life cycle of a virus can be divided into several overlapping stages, including attachment, cell entry, capsid loss, replication, assembly of viral particles, budding, and escape from a cell. Typically, the viral replication cycle begins with the attachment of a viral particle to specific receptors on the surface of host cells, which trigger virus entry by endocytosis, membrane fusion (virus envelope to cell membrane), and direct entry [2]. After internalization, the capsid is released into the cytoplasm and releases the viral genome, which replicates to make copies of the viral genome and is translated into viral proteins. In the endoplasmic reticulum (ER) and the Golgi complex, the assembly and maturation of viral proteins occur, which are then directed to the host cell membrane, where the progeny of viral particles are released [3].

Viruses are unable to reproduce outside the cell; therefore, they are obligate parasites. A significant proportion of viruses can cause viral diseases, which are infectious diseases and tumors [1]. The general patterns of viral infections correspond to those observed in any infectious processes. The main feature is the cytotropism and obligate intracellular parasitism of viruses, which makes them, in all respects (metabolically, energetically, and ecologically), dependent on the host cell. Typically, human DNA viruses replicate in the cell nucleus, while RNA viruses replicate in the cytoplasm. Since viruses use the natural metabolic pathways of their host cells to reproduce, they are difficult to eradicate without the use of drugs that are toxic to the host cells themselves. The most effective medical measures against viral infections are vaccinations, which create immunity to infection, and antiviral drugs, which selectively inhibit viral replication cycle.

Vaccination is the most effective way to prevent infectious diseases, being a cheap and effective way to prevent viral infections. There is a scientific consensus that vaccination is a reasonably safe and effective way to combat and eradicate infectious diseases. However, there are limits to its effectiveness, which include short term humoral response, mutation of the virus, no suitability for all individuals, etc. [4,5,6,7,8]. Sometimes the defenses do not work because the host’s immune system simply does not respond adequately or does not respond at all. Viruses can mutate and escape the antibodies produced by vaccines. Adverse reactions to vaccination and post-vaccination complications, although rarely occurring, can have serious consequences.

This requires the development of new approaches to the treatment of viral diseases.

## 2. Animal Venom and Their Components

Animal venoms are complex mixtures of substances of a protein and non-protein nature, which usually are called toxins. In this review, we confine ourselves to considering proteinaceous and peptidic toxins. Toxins are produced by many animal taxa. Snakes, scorpions, bees, and spiders are the best known and studied venomous terrestrial animals; and jellyfishes, anemones and cone snails are among marine toxic animals. Depending on the venomous species, the single venom may contain up to several hundred different toxins. Despite such a big diversity, the number of protein or peptide structural families represented in animal venoms is not very large. For example, about two dozen families can be found in snake venoms [9]. The most abundant families of snake toxins are phospholipases A2 (PLA2), three-finger toxins (3FTx), snake venom serine proteases (SVSP), and snake venom metalloprotease (SVMP). These are followed by further six protein families including cysteine rich secretory protein (CRiSP), Kunitz peptides (KUN), L-amino acid oxidases (LAO), natriuretic peptides (NP), C-type lectins (CTL), and disintegrins (DIS). The remaining families comprising hyaluronidases, phosphodiesterases, nerve growth factors, and some others can be considered as minor components.

Due to the high variability in venom composition, the classification of toxins present in scorpion venoms is not so well defined. In general, these are non-enzymatic and enzymatic toxins [10,11]. The non-enzymatic toxins include disulfide bridged proteins/peptides and non-disulfide bridged peptides (NDBPs). In turn, the disulfide bridged proteins/peptides are represented by different neurotoxin families affecting a variety of ion channels. The NDBPs make up major components of scorpion venoms and include so called antimicrobial peptides (AMPs). NDBPs consist of 13–56 amino acid residues and possess a very heterogeneous composition. Enzymatic toxins comprise phospholipases A2, serine and metalloproteinases, and hyaluronidases.

Due to low availability and limited impact on humans, spider venoms are not so well studied as those of snakes and scorpions. Spider venom toxins can be divided into four groups including peptide neurotoxins affecting various ion channels, proteins, and enzymes, as well as low molecular weight substances, and antimicrobial peptides, which are present in only a few spider families [12].

In bee venom, peptide melittin and phospholipase A2 are the most abundant components [13]. Melittin represents 50–60% of the venom dry weight and PLA2 accounts for around 10–12%. Other components can be considered as minor and include peptides apamin, mast cell-degranulation peptide (MCD), secapin, adolapin, as well as enzyme hyaluronidase.

Among marine animals, the most promising for pharmacology are venoms from mollusks of Conidae family. Their venoms are complex cocktails of toxic peptides, which are known as conotoxins or conopeptides [14]. The amino acid sequences of conopeptides vary greatly in length and number of disulfide bridges. Thus, conopressin-S is 9, while conkunitzin-S1 is 60 amino acid residues in length. Conopeptides affect numerous biological targets including voltage- and ligand-gated ion channels, G-protein coupled receptors as well as neurotransmitter transporters and produce diverse physiological effects.

So, such a great variability in composition and biological activity makes animal venoms an indispensable source of new compounds for both science and practice.

## 3. Antiviral Activity of Animal Toxins

Currently, antiviral drugs and vaccines are insufficient to control emerging and recurring viral diseases [15]. Thus, the discovery of new antiviral drugs is an urgent task. In general, antiviral therapy is the only approach to the specific treatment of viral infections that stops the viral replication cycle [16]. However, due to high genetic variability, viruses can quickly become resistant to antiviral treatment, especially RNA viruses [17]. In addition, antiviral therapy and long-term treatment can cause a number of side effects, including gastrointestinal disturbances, fatigue, headache, neuropathy, and liver toxicity [18].

According to the principle of action, antiviral drugs are divided into two groups: stimulating the immune system to attack viruses and attacking viruses directly. The drugs of the second group differ in the stage of the virus life cycle at which they are active: they prevent the entry of the virus into the cell, the replication of the virus inside the cell, and the release of copies of the virions from the cell. Interestingly, animal venoms also affect different stages of virus cycle.

### 3.1. Toxins with Antiviral Activity from Snake Venoms

The first mention of antiviral activity of snake venom dates back to the mid-sixties of the last century [19,20]. Detailed studies continued later and it was demonstrated that venoms of different snake species manifested activity against various species of viruses [21,22,23,24,25]. The majority of the snake species manifesting such activity were represented by those from Viperidae family. Thus, venoms of several snake species from Viperidae, Elapidae, and Crotalidae families were screened for antiviral activity against Sendai virus [21]. The venom of painted saw-scaled viper *Echis coloratus* was the most active, followed by the venoms of eastern saw-scaled viper *Echis carinatus sochureki* and cobras *Naja atra* and *Naja nigricollis nigricollis*. The venom of South American rattlesnake *Crotalus durissus terrificus* showed an inhibitory effect against measles virus [23]. More detailed study of this effect revealed that the infection was inhibited at the stages of adsorption and entry of the viral cycle [23]. The venoms of the western diamondback rattlesnake *Crotalus atrox*, rhombic night adder *Causus rhombeatus* and cobra *Naja melanoleuca* were studied for antiviral activity on adenoid 75 strain of human adenovirus type 5 and strain of human poliovirus type 1 [24]. All three venoms produced antiviral effects on both viruses although of different strength. The highest antiviral activity against both viruses was revealed for *N. melanoleuca* venom. *C. atrox* venom manifested higher antiviral activity against poliovirus and lowest one against adenovirus. These data show that snake venoms produce antiviral effects and may contain some compounds possessing antiviral activity. Interestingly, both enveloped (Sendai and measles viruses) and nonenveloped (adenovirus and poliovirus) viruses were affected. The genomes of the affected viruses were composed of a single-stranded RNA (poliovirus, Sendai, and measles viruses) or a double-stranded DNA (adenovirus).

The next steps logic is the identification and isolation of antiviral compounds from venoms. Thus, from the venom of *E. coloratus* manifesting antiviral activity against Sendai virus, the active factor was purified and called Echinhibin-1 [22]. Echinhibin-1 was shown to be a metalloprotease with a molecular weight of about 25 kDa. Virus pretreated with Echinhibin-1 and inoculated intranasally in mice produced no signs of infectivity. However, after the adsorption of Sendai virions onto washed erythrocytes, the treatment with Echinhibin-1 did not prevent its hemolytic activity, indicating that virus adsorption to the cells was disrupted by Echinhibin-1. It should be noted that the virus entry into the cell provides a very good possibility for therapeutic intervention. Noticeably, several venom components interfere with this stage. So, contortrostatin from the broad banded copperhead snake *Agkistrodon contortrix*, an Arg-Gly-Asp motif containing disintegrin, inhibited herpes simplex virus (HSV) entry into Chinese hamster ovary (CHO-K1) cells expressing HSV-1 glycoprotein D receptors and also into the primary cultures of human corneal fibroblasts [26]. Moreover, contortrostatin inhibited cell-to-cell fusion, and polykaryocyte formation in HSV infection. It was more active in naturally susceptible corneal fibroblasts than in CHO-K1 cells.

The other stages of virus replication cycle can be affected by snake venom components as well. So, L-amino acid oxidase from Stejneger’s pit viper *Trimeresurus stejnegeri* manifested dose dependent inhibition of human immunodeficiency virus type 1 (HIV-1) infection and replication [27]. The presence of catalase, a hydrogen peroxide scavenger, reduced anti-HIV-1 activity of oxidase, but the exogenous H_2_O_2_ alone did not display any anti-HIV-1 activity. In this case, the fusion of chronically HIV-1IIIB-infected H9 cells and uninfected C8166 cells in co-culture was not blocked, which suggests that virus binding to the host cell and absorption were not affected.

Another component possessing the antiviral activity was isolated from the venom of cobra *Naja naja oxiana* [25]. This was a protein with molecular mass of less than 10 kDa, which inhibited the infection of baby hamster kidney fibroblast cells (BHK-21 line) by Rabies virus CVS-11. As the author used the assay that allowed the virus to enter the cells, they suggest that the isolated protein inhibited the virus replication at the post entry stage. The main components of cobra venoms with molecular masses lower than 10 kDa are three finger cytotoxins and α-neurotoxins. The latter bind effectively and inhibit nicotinic acetylcholine receptors, which are considered also as receptors for Rabies virus [28].

It is quite surprising that among a large number of snake venom components, phospholipases A2 (PLA2) have been found to have the highest and most diverse antiviral activity. The first study of such PLA2 activity was conducted in 1999 by Fenard et al. [29]. It was found that two cobra venom PLA2s NmmCMIII and nigexine, as well as taipan PLA2 taipoxin very efficiently inhibited the infection of CD4+ HeLa cells (P4 cells) by HIV-1_BRU_. The study of the molecular mechanism of antiviral activity showed that enzymatic activity is not involved in the antiviral effect. PLA2s did not affect the virus binding to cells or syncytia formation, but the intracellular release of the viral capsid protein was inhibited. So, viral entry into cells before virion uncoating was blocked by PLA2s. The four most active venom PLA2s were found to bind with high affinities to host cells, so the involvement of PLA2-cell interaction cannot be excluded from antiviral effect. The study of anti-HIV activity of snake venom PLA2s was updated on recently appeared highly pathogenic HIV-1 MvP-899, HIV-1 Zmb, and HIV-2 EHO strains, as well as infectious molecular clones K3016 and AD8 [30]. Several snake venom PLA2s including dimeric PLA2s HDP-1 and HDP-2 from Nikolskii’s viper *Vipera nikolskii* were used in this study. It was found that HDP-2 strongly inhibited all HIV variants. It was also found that the studied PLA2s inhibited syncytium formation between chronically HIV-infected cells and healthy CD4-positive cells and blocked HIV binding to cells. The dimeric PLA2s manifested the highest virucidal and anti-HIV activity, which, in contrast to the previous study [29], depended on their catalytic activity.

A number of papers have been dedicated to the study of antiviral activity of crotoxin and other PLA2s from the venoms of snakes of the *Crotalus* genus.

Thus, antiviral activity of South American rattlesnake *C. durissus terrificus* PLA2s against Dengue virus (DENV, strain NGC) and yellow fever virus (YFV, strain 17D) was studied in VERO E6 and C6/36 cell lines [31]. It was found that crude rattlesnake venoms (yellow and white), crotoxin, PLA2-CB, and PLA2-IC strongly inhibited YFV and DENV replication in VERO E6 cells. The highest effects on viral replication cycle were found in the virucidal, pre-treatment, and adsorption assays, which means that the initial steps of the replication cycle were inhibited. The result obtained suggests that phospholipolytic activity is involved in the antiviral effect. This hypothesis was confirmed in the study of enzymatically inactive PLA2 BthTX-I from *Bothrops jararacussu* venom. Although BthTX-I manifested some antiviral activity, it was several orders of magnitude lower than that of crotoxin, PLA2-CB, and PLA2-IC [31]. In addition to DENV and YFV, PLA2-CB and crotoxin showed high antiviral activity against Dengue virus type 2, Rocio virus, Mayaro virus, and Oropouche virus [32], which all are enveloped viruses. The non-enveloped Coxsackie B5 virus was not inactivated. A further study of the molecular mechanism of antiviral activity for PLA2s from *C. durissus terrificus* showed that their effects were realized via the cleavage of glycerophospholipids at the virus lipid bilayer envelope. This resulted in a partial exposure of genomic RNA and a destabilization of the E proteins on the virion surface, leading to the virus inactivation [32]. The action of PLA2-CB and crotoxin on the enveloped viruses and absence of the effects on non-enveloped virus support this conclusion.

In addition to the viruses mentioned above, hepatitis C virus (HCV) was also affected by crotoxin and by its subunits crotapotin and PLA2-CB [33]. The HCVcc JFH-1 strain was used to infect human hepatoma-derived Huh 7.5 cells. To determine the affected stage of the virus life cycle, toxins were added to the cells at different times. It was found that HCV entry and replication, but no HCV release, were inhibited by PLA2-CB. Crotoxin reduced virus entry and release but no effect on replication was observed. The only stage inhibited by crotapotin was HCV release. These data showed that snake toxins can produce multiple antiviral effects on the HCV life cycle [33].

An array of viruses studied has been expanded further and antiviral activity of PLA2-CB was checked against the Zika virus (ZIKV) and the chikungunya virus (CHIKV). The investigation of two recombinant PLA2-CB isoforms showed that they exerted virucidal effect on ZIKV and CHIKV as well as on DENV and YFV [34]. A more detailed study of native PLA2-CB activity against CHIKV was carried out using BHK-21 cells [35]. It was found that PLA2-CB strongly hinder the virus entry into the cell by reducing adsorption and post-attachment stages. In addition, PLA2-CB manifested a noticeable activity at the post-entry stages of CHIKV replicative cycle. Spectroscopy measurements and docking calculations suggested PLA2-CB interaction with CHIKV glycoproteins, and this interaction may block the binding of CHIKV virions to the host cells [35].

The results discussed above demonstrated that phospholipolytic activity of PLA2s is involved in their antiviral activity. However, enzymatic activity may not be absolutely necessary for antiviral activity, and there are some data that show that PLA2s that do not hydrolyze lipids inhibit the replication of viruses. So, two PLA2s isolated from *B. leucurus* venom, enzymatically inactive BlK-PLA2, containing Lys49 in the active center, and enzymatically active BlD-PLA2 with Asp49 in active center, were examined for antiviral activity against DENV [36]. For this purpose, PLA2s were added to the rhesus monkey kidney epithelial cells (LLC-MK2 line) and after that the cells were infected with DENV. In cells treated with PLA2s, a significant reduction in the number of DENV RNA copies was observed. No significant difference in antiviral activity between BlK- and BlD-PLA2 was found. The PLA2s exerted no significant inhibitory effect on the viral replication cycle if they were added to the cells after viral adsorption [36]. These results indicate that PLA2s somehow affect the cell membrane, one of the ways is that they may block the virus receptor on the cell surface.

Different results concerning antiviral activity of enzymatically active and inactive PLA2s were obtained at the study of the antiviral action of Mt-I (catalytically active PLA2) and Mt-II (catalytically inactive variant) isolated from the venom of pit viper *B. asper* [37]. Among a large set of studied viruses, enveloped YFV and DENV were strongly inactivated by PLA2s, in contrast to non-enveloped Sabin virus or enveloped Influenza A, Herpes simplex 1 and 2, and Vesicular Stomatitis viruses, which were only slightly affected by toxins. The antiviral effect against highly susceptible DENV was 1000-fold stronger with Mt-I than Mt-II. The chemical inactivation of Mt-I strongly diminished its antiviral activity. Mt-I exerted a direct virucidal effect being active prior to infection, and did not affect the host cells. It was found that Mt-I produced a much stronger effect on DENV2 propagated in mosquito cells than on human cell-propagated virus [37]. It means that differences in the composition of envelope membrane may be important for the virucidal action of PLA_2_ enzymes.

The importance of the viral envelop membrane composition for the antiviral PLA2 activity was further confirmed in the work of Chen et al. [38]. They have studied the effects of CM-II isoform of secreted PLA2 from cobra *N. mossambica mossambica* (CM-II-PLA2) on a set of viruses in which the lipid bilayers of envelopes were acquired from either budding through the endoplasmic reticulum (HCV, DENV, and Japanese encephalitis virus (JEV)) or through the plasma membrane (Sindbis virus, influenza virus, and Sendai virus) or trans-Golgi network (HSV). It should be noted that the phospholipid contents of viruses budding through various membranes are different. It was found that CM-II-PLA2 possessed potent virucidal activity against HCV, DENV, and JEV that bud through the endoplasmic reticulum, but several orders of magnitude lower activity against viruses that bud through the plasma membrane or trans-Golgi network. Moreover, CM-II-PLA2 did not significantly affect the plasmatic membrane of Huh7it-1 cells used in that study [38].

It should be noted that SARS-CoV-2, which caused recently the COVID-19 pandemic, is budding through the endoplasmic reticulum [39]. In accordance with the above results, SARS-CoV-2 was highly susceptible to the action of PLA2s [40]. A series of PLA2s from the snake venoms were tested for antiviral activity against SARS-CoV-2 and it was found that dimeric PLA2s from the viper *V. nikolskii* showed especially potent virucidal effects and inhibited SARS-CoV-2 spike glycoprotein-mediated cell–cell fusion. The PLA2s effects were related to their phospholipolytic activity. PLA2s inhibited binding both of the receptor-binding domain of the SARS-CoV-2 glycoprotein S and of an antibody against ACE2 to 293T/ACE2 cells. It was also found that *V. nikolskii* PLA2 was capable to bind both ACE2 and receptor-binding domain of the glycoprotein S [40]. These data indicate that PLA2s may inhibit the virus binding to its receptor on the host cell membrane.

Summarizing data for snake venom PLA2 antiviral effects, one can say that these toxins efficiently inhibit the reproduction of viruses especially those budding through the endoplasmic reticulum. The most affected stages of viral replication cycle are binding to and virus entry into the host cells; however, some post-entry stages can be affected as well. Thus, PLA2s exert multiple effects on the virus replication cycle. More detailed information about possible molecular mechanisms of PLA2 antiviral effects and their proviral role can be found in recent reviews [41,42].

### 3.2. Antiviral Peptides Based on Snake Toxins

Although some snake toxins have strong antiviral activity, they are unlikely to be used as such to treat viral infections. One of the ways for the practical use of toxins is the design of antiviral peptides with the toxin as their basis; another way is an identification of antiviral peptides in snake venoms.

As to the design of antiviral peptides, the antiviral effects against SARS-CoV-2 of the covalently linked dimers representing the C-terminal fragments of enzymatically inactive PLA2 BthTX-I, which was discussed above, were reported [43]. Although the effective concentrations were very high with EC_50_ being in the range of 28–65 µM, this work showed the feasibility for the design of antiviral peptides based on snake toxins. Moreover, it was shown that the designed peptides possessed inhibitory activity against the Papain-like protease (PL^pro^) of SARS-CoV-2, the protease involved in the infection cycle. This finding opens the way to the structure–activity relationship (SAR)-based construction of effective PL^pro^ inhibitors, i.e., antiviral drugs.

Snake venoms contain mainly protein toxins; however, some peptides are also present. The most abundant are bradykinin-potentiating and natriuretic peptides for which no antiviral activity have been reported so far. However, there is a group of antimicrobial defense peptides, called cathelicidins, which are found in snake venoms [44]. Thus, cathelicidin BF-30, a 30 amino acid residue peptide, was isolated from the krait *Bungarus fasciatus* venom [45]. It was reported that BF-30 inhibited the influenza virus strains H1N1, H3N2, and the oseltamivir-resistant strain H1N1 [46]. The EC_50_ values were in the range of about 5–20 μM, while cytotoxic concentrations of BF-30 to Madin–Darby canine kidney (MDCK) cells were higher with CC_50_ being of 67.7 μM. Molecular mechanism studies have shown that BF-30 affected only the virus entry stage. The peptide also manifested some in vivo activity by enhancement of infected mice survival [46].

Using BF-30 as a template, a tryptophan and lysine/arginine-rich cationic peptide VKRWKKWRWKWKKWV-NH_2_ was designed, named ZY13 [47], and was shown to affect ZIKV infection in vitro and in vivo [48]. Using qRT-PCR, it was found that ZY13 inhibited ZIKV replication in human U251 and monkey Vero cells with IC_50_ in the range of 1–2 µM, while the peptide was less effective against DENV-2. ZY13 manifested virucidal activity and decreased the production of infectious virions. ZY13 also reinforced the host antiviral immunity via AXL-SOCS (suppressor of cytokine signaling protein) pathway and efficiently restrained ZIKV infection in vivo by improvement of the growth defects in ZIKV-infected mouse pups [48].

### 3.3. Toxins with Antiviral Activity from Scorpion Venoms

Compared to snake venoms, in scorpion venoms, the most abundant are toxins of lower molecular weight not exceeding in general 10 kDa, which are mainly represented by neurotoxins and antimicrobial peptides. The proteins with molecular masses above 10 kDa are enzymes including PLA2s, lyzozymes, hyaluronidases, and proteases.

Interestingly, similarly to snake toxins, antiviral activity was demonstrated for PLA2 from scorpion *Liocheles australasiae* venom, named LaPLA2-1 [49]. It has a heterodimeric structure and N-glycosylated at the N-terminal region. On Huh7it-1 human liver cell line, it manifested high virucidal activity against HCV, DENV, and JEV, but not against HSV-1. At post-entry treatment, this PLA2 exerted practically no effects on viruses. In the presence of manoalide, the PLA2 inhibitor, a strong decrease in the LaPLA2-1 virucidal activity was observed [49]. This suggests that phospholipolytic activity of LaPLA2-1 may be involved in its antiviral activity. Thus, scorpion PLA2 is similar to snake PLA2s in manifestation of antiviral activity.

A large proportion of toxins in scorpion venoms is represented by antimicrobial peptides (AMPs), a group of peptides that are quite heterogeneous in amino acid sequences. As the name implies, these peptides exhibit toxicity to microorganisms, and some of them have antiviral activity.

So, AMP called mucroporin was identified in the cDNA library from the venom gland of scorpion *Lychas mucronatus* [50]. Based on the protein mucroporin sequence, mucroporin-M1 (LFRLIKSLIKRLVSAFK) was designed, and both peptides were tested for antiviral activity against measles, SARS-CoV and influenza H5N1 viruses, enveloped RNA viruses [51]. While mucroporin was inactive, mucroporin-M1 showed activity against all three viruses in micromolar range. The data obtained suggested that mucroporin-M1 inhibited measles, SARS-CoV, and influenza H5N1 viruses by direct virucidal action. The decrease in infectivity of the viruses by mucroporin-M1 may result from the peptide binding to the virus envelope by surface charge interactions. Interestingly, no activity against vaccinia virus, an enveloped DNA virus, was observed [51].

Several putative AMPs were identified in a venom gland cDNA library from the scorpion *Euscorpiops validus*; the peptides were synthesized and screened for antiviral activity against HSV-1 [52]. One peptide comprising 13 amino acid residues (LWGEIWNTVKGLI) and called Eval418 showed high activity in protection of Vero cells against HSV-1. A time of addition experiment showed that Eval418 dose-dependently inhibited HSV-1 attachment to host cells, but exerted only a weak effect against established HSV-1 infection. To enhance the post-entry activity, the histidine residues were introduced in the sequence of Eval418. The peptide containing five histidine residues (LWHHGEIWHNTVHHKGLI) manifested the highest antiviral effects and reduced the infectivity of the extracellular and intracellular HSV-1 by 82% and 78%, respectively [52]. The intracellular location of the peptide was confirmed by confocal microscopy and flow cytometry.

A series of scorpion AMPs manifested antiviral activity against DENV, ZIKV, and HCV [53,54,55,56].

Thus, new AMP (IFKAIWSGIKSLF) called Hp1090 was chosen based on the cDNA library from the venom gland of scorpion *Heterometrus petersii*. The peptide was synthesized and tested for antiviral activity against HCV [53]. It was found that Hp1090 inhibited HCV infection with an IC_50_ of 5.0 μM. It manifested virucidal activity by the permeabilization of phospholipid membranes in the virion envelope [53].

A recombinant peptide rEv37 (molecular mass of 8503.96 Da) from the scorpion *Euscorpiops validus* inhibited DENV-2, HCV, ZIKV, and HSV-1 infections but had no effect on the Sendai virus and adenovirus [55]. rEv37 manifested very low virucidal activity and only moderately prevented infection after the pretreatment of the Huh7cells. However, it strongly reduced DENV-2 infection at the post-entry cycles of replication. More detailed studies indicated that rEv37 blocked the release of the viral genome from the endosome to the cytoplasm, thus impairing the viral late entry [55]. So, rEv37 manifested a broad spectrum of antiviral effects affecting the viruses undergoing low pH-dependent activation of fusion during entry into host cells.

A naturally occurring antiviral peptide, Smp76, containing 76 amino acids with 6 cysteine residues was purified from *Scorpio maurus palmatus* venom and showed high antiviral activity against HCV (JFH1, genotype 2a) and DENV (Trinidad 1751, type 2) [52]. Smp76 did not affect the viral replication cycle, but inhibited HCV infection and suppressed secondary infection, by inactivating extra-cellular infectious particles. Detailed studies showed that Smp76 suppressed the established viral infection, and its effect was similar to that of interferon β [54]. The molecular mechanism involved upregulation of the interferon β expression by activating interferon regulatory transcription factor 3 (IRF3) phosphorylation. The mechanism of Smp76 effects is substantially different from that of traditional virucidal AMPs and shows that AMPs can function as immunomodulators [54].

Another scorpion peptide demonstrating antiviral activity against HCV was a scorpion defensin, BmKDfsin3, derived from the venom of *Mesobuthus martensii* and containing 38 amino acid residues with 6 cysteine residues forming 3 disulfide bonds [57]. It affected viral attachment and post-entry stages in HCV life cycle. Further studies showed that BmKDfsin3 entered into cells and inhibited activation of p38 mitogen-activated protein kinase, which suppressed HCV replication [57].

Immunodeficiency viruses (simian (SIV) and human immunodeficiency virus (HIV)) attack immune cells and eradication of these viruses represents a special task, and AMPs from scorpion venoms show some hints how to combat this virus. Thus, the transcriptome analysis of cDNA libraries from venom glands of scorpions *Tityus obscurus*, *Opisthacanthus cayaporum*, and *Hadrurus gertschi* revealed several putative AMPs that were synthesized and checked for antiviral activity against SIV [58]. The *T. obscurus* scorpion-derived peptide (FFGTLFKLGSKLIPGVMKLFSKKKER) manifested the highest activity against SIV replication in the HUT-78 cells and primary human leukocytes. The primary human leukocytes treated with the peptide and infected with SIV demonstrated synthesis of proinflammatory and anti-inflammatory cytokines, which could be involved in cell defense mechanisms [58].

A series of scorpion AMPs were screened for anti-HIV activities [59]. It included mucroporin, mucroporin-M1, and mucroporin-S1 from *L. mucronatus*, BmKn2 (FIGAIARLLSKIF) from the venom of *M. martensii,* and Kn2-7 (FIKRIARLLRKIF) designed based on the sequence of BmKn2. Kn2-7 manifested the highest anti-HIV-1 activity with EC_50_ value of 1.65 µM. Kn2-7 inhibited the viruses of a standard reference panel of HIV-1 subtype B showing comparable antiviral potencies against 13 strains of HIV-1 subtype B including 12 R5-tropic (6535, QH0692, SC422661, PVO, TRO, AC10, PHPA4259, THRO4156, REJO4551, TRJO4551, WITO4160, and CAAN5342) and 1 X4-tropic (NL4-3). It was found that anti-HIV-1 activity correlated with a direct binding of Kn2-7 to HIV-1 envelope [59].

The initial stage of SARS-CoV-2 entry in the host cell is the interaction of virus spike glycoprotein S with the ACE2 receptor on cell membrane. To find the substances with putative anti-SARS-CoV-2 activity, the docking analysis of some scorpion AMPs binding to receptor-binding domain of glycoprotein S was carried out [60]. HP 1090 from *H. petersii* as well as meucin-13 (IFGAIAGLLKNIF-NH_2_) and meucin-18 (FFGHLFKLATKIIPSLFQ) from *M. eupeus* were used for docking experiments. Meucin-18 (FFGHLFKLATKIIPSLFQ) showed better interaction with the domain than other peptides. After the study of several meucin-18 analogues, it was found that the peptide with A9T substitution had more effective interaction with the domain than the meucin-18 itself [60]. Thus, this peptide (FFGHLFKLTTKIIPSLFQ) should be further checked for antiviral activity against SARS-CoV-2.

### 3.4. Toxins with Antiviral Activity from Bee and Spider Venoms

The data about antiviral activities of components from bee and spider venoms are not so numerous as that for snake and scorpion venoms. Although the composition of spider venoms is quite complex, the composition of bee venom is not so diverse [13]. As mentioned above, the main components are peptide melittin and PLA2. The antimicrobial activity of bee venom is well known [61], and its antiviral activity against Papillomaviruses [62], HSV [63], HCV [64], and some others has been demonstrated.

Being the most abundant, melittin is a main antiviral component in bee venom. While being cytotoxic to Vero cells, melittin showed a high antiviral activity against Arenavirus Junin, HSV-1, and HSV-2 [65]. It affected both viruses via inhibition of virus multiplication, adsorption, and penetration as well as Na^+^ and K^+^ pumps of the host cell [66]. This peptide manifested toxicity to a wide range of viruses significantly inhibiting the replication of enveloped viruses such as Influenza A virus (PR8), Vesicular Stomatitis Virus (VSV), Respiratory Syncytial Virus (RSV), and HSV [63]. Moreover, non-enveloped viruses Enterovirus-71 (EV-71) and Coxsackie Virus (H3) were also inhibited be melittin. Its antiviral effects are mainly realized through a virucidal mechanism [63]. The study of the melittin effect on HIV-1 replication and gene expression in acutely infected cells showed the virus inhibition in a dose-dependent manner, with ID50 values in the range 0.9–1.5 µM [67]. Decreased levels of Gag antigen and HIV-1 mRNAs were revealed during detailed analysis of the melittin effect on the cell-associated virus production. The observed HIV-1 inhibition by melittin was realized through the suppression of HIV-1 gene expression [67].

Although melittin manifested good antiviral activity, it was quite toxic to host cells. To overcome this problem, its synthetic analogues were designed. One of them called Hecate, inhibited HSV-1 [68]. Cell fusion induced by HSV-1 syncytial mutants was completely inhibited by Hecate. This peptide manifested no toxic effects on eukaryotic cells and inhibited virus-induced cell fusion and virus spread without affecting any other viral functions [68]. To develop further antiviral compounds based on melittin, Hecate’s N-terminus region was modified and the effects of the derivatives obtained were studied against the HCV. Gallic acid-conjugated Hecate was the most efficient and inhibited between 50% and 99% of all major steps of the HCV infectious cycle [69]. The conjugate mechanism of action included a balanced lipid interaction with the viral envelope and lipid droplets, as well as dsRNA intercalation. This conjugate may also affect other ssRNA viruses and those with a lipid-dependent cycle [69].

PLA2 from the bee venom was the one manifesting the highest activity against HIV [29]. However, it possessed high cytotoxicity as well. In the search for the site responsible for antiviral activity, 12 synthetic peptides derived from PLA2 have been studied for the blocking of the HIV-1 infection [70]. The p3bv peptide, comprising amino acids 21–35 of PLA2 inhibited the replication of T-tropic HIV-1 isolates but was inactive on M-tropic HIV-1 isolates. The peptide prevented the cell–cell fusion mediated by the T-tropic HIV-1 envelope. Further studies indicated that p3bv inhibited the replication of T-tropic HIV-1 strains by interacting with CXCR4. It should be mentioned that the p3bv mechanism of action was different from that of PLA2, which inhibited replication of both T-tropic and M-tropic isolates [29].

Information about the antiviral activity of spider toxins is quite limited. So, in the *Alopecosa nagpag* spider venom, a defense peptide named An1a was identified and its antiviral activity against DENV2 was studied [71]. It was found that An1a is a DENV2 NS2B-NS3 protease inhibitor. An1a also blocked ZIKV infection by inhibiting the ZIKV NS2B-NS3 protease. These data show that spider venom is a potential source of antiviral precursor molecules [71].

Such poor representation of compounds with antiviral activity in spider venoms may soon be changed with the use of a new methodology that consists of a multiomics approach involving proteomics, peptidomics, and transcriptomics analyses allied to in silico predictions of antibacterial, antifungal, antiviral, and anticancer activities. Through the application of this strategy for the venom of *Acanthoscurria rondoniae,* it was suggested that 7 cysteine-rich peptides families (from a total of 92,889 venom gland transcripts) may have potential antimicrobial or antiviral activities [72].

### 3.5. Toxins with Antiviral Activity from Marine Organisms

One of the simplest aquatic organisms is cyanobacteria (blue-green algae). A 11-kDa protein cyanovirin-N, which irreversibly inactivated both laboratory strains and primary isolates of HIV-1 and HIV-2 at low nanomolar concentrations, was isolated from *Nostoc ellipsosporum*. This protein also aborted cell-to-cell fusion and transmission of HIV-1 infection [73]. Its antiviral activity has been attributed to a tight binding to the viral envelope glycoprotein gp120.

A great potential for obtaining antibacterial and antiviral drugs was stated in [74] after the isolation and structural and functional characterization of the 36-membered peptide asteropine A from a sea sponge *Asteropus simplex*, which competitively inhibited different sialidases.

Until recently, marine gastropod venoms were not considered as sources of antiviral compounds. However, the screening of several cone snail venoms revealed the inhibitory activity of χ-conopeptide MrIA (χ-MrIA) from the venom of the snail *Conus marmoreus* against serotype 2 Dengue virus NS2B-NS3 protease [75]. Moreover, this effect was attributed to a short fragment of MrIA that made possible the design and synthesis of a more potent and stable cyclic analogue with high potency (inhibitory constant of 2.2 μM), which could be considered as a good lead for Dengue antivirus drug development.

One of the most ancient representatives of the marine animal world that have survived to this day—horseshoe crabs *Tachypleus tridentatus* and *Limulus Polyphemus*—produced the polypeptides tachyplesins and polyphemusins, on the basis of which small peptide analogues (T22 and T134) with antiviral activity to HIV-1 were obtained [76,77]. For these peptides, the importance of arginine residues for antiviral activity, attributed to antagonism towards the CXCR4 coreceptor, was revealed [78,79].

More recently, another compound was isolated from an unusual sea creature—cuttlefish *Sepia prashadi*. It turned out to be a small peptide posterior salivary gland (PSG) toxin that showed antiviral activity against the avian New Castle disease virus in the Zebrafish embryo model [80].

Finally, the study of the antiretroviral activity of the PLA2 from *Pterois volitans* red lionfish venom showed the significant inhibition of simian retrovirus serotype-2 (SRV2) culture in vitro. It gives a certain potential for this protein to become an anti-HIV substance [81].

The data about antiviral activity of the animal venom peptides and proteins as well as about the viruses affected by these compounds are summarized in Table 1.

## 4. Problems and Prospects for Drug Development on the Basis of Animal Venoms

The above data show that a lot of compounds from animal venoms possess antiviral activity. In some papers, it was declared that the found compounds can be used as drugs. Is it really possible? It is clear that toxins with high antiviral activity, as such, also have high toxicity. However, there are no data about the toxicities of the discussed compounds to humans, while some of them were used for the treatment of viral infection in rodent models [46]. High toxicity is inherent mainly to components of snake venoms—PLA2, metalloproteinase, disintegrin, etc. All these compounds manifest antiviral activity at concentrations that are several orders of magnitude lower that those producing cytotoxicity. Although they manifest antiviral activity at very low concentrations, they hardly can be applied as drugs. The main disadvantages of these compounds are toxicity, immunogenicity, and instability in the organism. The possible solution of these problem may be the identification of active sites responsible for antiviral activity, if any exists, and synthesis of short peptide fragments based on the identified amino acid sequences. As discussed above, this approach was used for enzymatically inactive PLA2 BthTX-I [43] and resulted in the discovery of peptides possessing antiviral activity, although not high.

More promising is the use of animal venom peptides with antiviral activity [82]. Peptide toxins have a range of advantages such as a high selectivity of action and a high affinity to their biological targets, low immunogenicity, ample opportunities for structure optimization, and cost-effective production, which make them good candidates for drug leads. A number of peptides with antiviral activity are discussed above. However, there are still some problems that should be solved. One of them is stability of the peptide drugs in the organism. This problem can be solved by the optimization of the amino acid sequence with the incorporation of unnatural amino acids including β-amino acids or the modification of peptide bonds. Further, to improve the pharmacokinetic and biodistribution of the peptides, they can be chemically conjugated to the protein carrier (e.g., albumin) or polymer (e.g., polyethylene glycol) [83,84]. Another approach is acylation with a fatty acid, which promotes the formation of a self-associated multimolecular complex [85]. The design of low molecular weight mimetics can also be used to solve this problem. This approach has already been applied for the design of anti-HIV compounds [86]. The alternative approach is encapsulation of the peptides in nanomaterials—nanogels, liposomes, etc. For example, such approach is already used to increase bioavailability and provide a long release period of proteins and peptides encapsulated in the microspheres [87]. Another problem that may be encountered in the design of peptide-based antiviral drugs is low selectivity. Some peptides discussed above manifested high antiviral activity and low cytotoxicity to the host cells. However, animal or human organisms contain a huge variety of different cells, which can vary greatly in sensitivity to antiviral peptides. This will require careful toxicological studies and their outcome is unpredictable. Therefore, a large amount of additional research is required to convert animal toxins into antiviral drugs.

## 5. Conclusions

Viruses infect all types of organisms, causing viral diseases that are very common in humans. Since viruses use the metabolic pathways of their host cells to replicate, they are difficult to eradicate without affecting the cells. The most effective measures against viral infections are vaccinations and antiviral drugs, which selectively inhibit the viral replication cycle. Both methods have disadvantages, which requires the development of new approaches to the treatment of viral diseases. In the study of animal venoms, it was found that, in addition to toxicity, venoms exhibit other types of biological activity, including an antiviral one, the first mention of which dates back to the middle of the last century. However, detailed studies of venom antiviral activity have been conducted over the past 15 years. The COVID-19 pandemic has reinforced these studies. To date, a number of compounds with antiviral activity have been identified in venoms. Some of them are very active and can be considered as the basis for antiviral drugs. The review of the available literature shows that studies of antiviral activity of animal venoms conducted in recent years resulted in the discovery of a number of compounds with high antiviral activity. Their antiviral effects include different molecular mechanisms. Some of the compounds have good prospects for the creation of antiviral drugs; however, there are several problems that should be solved to enable the transformation of toxins into drugs.

## Figures and Tables

**Table 1:** The animal venom peptides and proteins with antiviral activity.

Virus Species	Venom Protein	Venomous Animal Species	Reference
Arenavirus Junin	Melittin	Honey bee *Apis mellifera*	[65]
Avian New Castle disease virus	Posterior salivary gland (PSG) toxin	Cuttlefish *Sepia prashadi*	[80]
Chikungunya virus (CHIKV)	PLA2-CB	South American rattlesnake *Crotalus durissus terrificus*	[34]
Coxsackie Virus (H3)	Melittin	Honey bee *Apis mellifera*	[63]
Dengue virus (DENV)	PLA2s: crotoxin, PLA2-CB and PLA2-IC	South American rattlesnake *Crotalus durissus terrificus*	[31]
	PLA2 BlD-PLA2, containing Asp49	Whitetail lancehead *Bothrops leucurus*	[36]
	PLA2 BlK-PLA2, containing Lys49	Whitetail lancehead *Bothrops leucurus*	[36]
	PLA2s: Mt-I (catalytically active PLA2) and Mt-II (catalytically inactive variant)	Terciopelo *Bothrops asper*	[37]
	PLA2 CM-II isoform (CM-II-PLA2)	Cobra *Naja mossambica mossambica*	[38]
	PLA2 LaPLA2-1	Scorpion *Liocheles australasiae*	[49]
Dengue virus type 2 (DENV-2)	PLA2s: PLA2-CB and crotoxin	South American rattlesnake *Crotalus durissus terrificus*	[32]
	A recombinant peptide rEv37	Scorpion *Euscorpiops validus*	[55]
	Antiviral peptide Smp76	Scorpion *Scorpio maurus palmatus*	[56]
	Peptide An1a	Spider *Alopecosa nagpag*	[71].
	χ-Conopeptide MrIA (χ-MrIA)	Marine snail *Conus marmoreus*	[75]
Enterovirus-71 (EV-71)	Melittin	Honey bee *Apis mellifera*	[63]
Hepatitis C virus (HCV)	PLA2 CM-II isoform (CM-II-PLA2)	Cobra *Naja mossambica mossambica*	[38]
	PLA2 LaPLA2-1	Scorpion *Liocheles australasiae*	[49]
	AMP Hp1090 IFKAIWSGIKSLF	Scorpion *Heterometrus petersii*	[53]
	A recombinant peptide rEv37	Scorpion *Euscorpiops validus*	[55]
	Defensin BmKDfsin3	Scorpion *Mesobuthus martensii* Karsch	[57]
HCV, JFH-1 strain	Crotoxin and its subunits crotapotin and PLA2-CB	South American rattlesnake *Crotalus durissus terrificus*	[33]
HCV, JFH1, genotype 2a	Antiviral peptide Smp76	Scorpion *Scorpio maurus palmatus*	[56]
Human immunodeficiency virus type 1 (HIV-1)	L-amino acid oxidase	Stejneger’s pit viper *Trimeresurus stejnegeri*	[27]
	PLA2 from the bee venom	Honey bee *Apis mellifera*	[29]
	Peptide analogue T22 of the polypeptide tachyplesin	Horseshoe crab *Tachypleus tridentatus*	[76]
	Peptide analogue T134 of the polypeptide polyphemusin	Horseshoe crab *Limulus polyphemus*	[77]
HIV-1_BRU_	PLA2 NmmCMIII	Cobra *Naja mossambica mossambica*	[29]
	PLA2 nigexine	Cobra *Naja nigricollis*	[29]
	PLA2 taipoxin	Taipan *Oxyuranus scutellatus*	[29]
HIV-1 subtype B including R5-tropic (6535, QH0692, SC422661, PVO, TRO, AC10, PHPA4259, THRO4156, REJO4551, TRJO4551, WITO4160, CAAN5342), and X4-tropic (NL4-3)	Peptide Kn2-7 (FIKRIARLLRKIF) designed based on the sequence of peptide BmKn2	Scorpion *Mesobuthus martensii*	[59]
HIV-1 and HIV-2	Cyanovirin-N	Cyanobacteria (blue-green algae) *Nostoc ellipsosporum*	[73]
HIV-1 MvP-899, HIV-1 Zmb, HIV-2 EHO, and infectious molecular clones K3016 and AD8	Dimeric PLA2s HDP-1 and HDP-2	Nikolskii’s viper *Vipera nikolskii*	[30]
Herpes simplex virus (HSV)	Arg-Gly-Asp motif containing disintegrin contortrostatin	Broad banded copperhead snake *Agkistrodon contortrix*	[26]
HSV-1	Eval418 peptide (LWGEIWNTVKGLI) and analogue (LWHHGEIWHNTVHHKGLI)	Scorpion *Euscorpiops validus*	[52]
	A recombinant peptide rEv37	Scorpion *Euscorpiops validus*	[55]
HSV-1 and HSV-2	Melittin	Honey bee *Apis mellifera*	[63,65]
Influenza virus strains H1N1, H3N2, and the oseltamivir-resistant strain H1N1	Cathelicidin BF-30	Krait *Bungarus fasciatus* venom	[46]
Influenza H5N1	Mucroporin-M1 (LFRLIKSLIKRLVSAFK)	Scorpion *Lychas mucronatus*	[51]
Influenza A virus (PR8)	Melittin	Honey bee *Apis mellifera*	[63]
Japanese encephalitis virus (JEV)	PLA2 CM-II isoform (CM-II-PLA2)	Cobra *Naja mossambica mossambica*	[38]
	PLA2 LaPLA2-1	Scorpion *Liocheles australasiae*	[49]
Mayaro virus	PLA2s: PLA2-CB and crotoxin	South American rattlesnake *Crotalus durissus terrificus*	[32]
Measles viruse	Mucroporin-M1 (LFRLIKSLIKRLVSAFK)	Scorpion *Lychas mucronatus*	[51]
Oropouche virus	PLA2s: PLA2-CB and crotoxin	South American rattlesnake *Crotalus durissus terrificus*	[32]
Rabies virus CVS-11	A protein with molecular mass of less than 10 kDa	Cobra *Naja naja oxiana*	[25]
Respiratory Syncytial Virus (RSV)	Melittin	Honey bee *Apis mellifera*	[63]
Rocio virus	PLA2s: PLA2-CB and crotoxin	South American rattlesnake *Crotalus durissus terrificus*	[32]
SARS-CoV	Mucroporin-M1 (LFRLIKSLIKRLVSAFK)	Scorpion *Lychas mucronatus*	[51]
SARS-CoV-2	Dimeric PLA2s HDP-1 and HDP-2	Nikolskii’s viper *Vipera nikolskii*	[40]
Sendai virus	Metalloprotease Echinhibin-1	Saw-scaled viper *Echis coloratus*	[22]
Simian immunodeficiency virus (SIV)	Peptide (FFGTLFKLGSKLIPGVMKLFSKKKER)	Scorpion *Tityus obscurus*	[58]
Simian retrovirus serotype-2 (SRV2)	PLA2	Red lionfish *Pterois volitans*	[81]
Vesicular Stomatitis Virus (VSV)	Melittin	Honey bee *Apis mellifera*	[63]
Yellow fever virus (YFV)	PLA2s: Mt-I (catalytically active PLA2) and Mt-II (catalytically inactive variant)	Terciopelo *Bothrops asper*	[37]
YFV, strain 17D	PLA2s: crotoxin, PLA2-CB, and PLA2-IC	South American rattlesnake *Crotalus durissus terrificus*	[31]
Zika virus (ZIKV)	PLA2-CB	South American rattlesnake *Crotalus durissus terrificus*	[34]
	A recombinant peptide rEv37	Scorpion *Euscorpiops validus*	[55]
	Peptide An1a	Spider *Alopecosa nagpag*	[71]

## Data Availability

Not applicable.
